# Annotations

**Published:** 1894-06-30

**Authors:** 


### Annotations.

### Guardians and Nurse Gillespies.

Now that the natural indignation aroused by the conduct of Nurse Gillespie in the Poor Law School at Brentwood has somewhat calmed down, we may consider what practical results can be educed from its exposure for the advantage of the unfortunate children. Many people have said that Nurse Gillespie must have been a fiend in human form, and having given utterance to that sentiment their interest in the subject has ceased. But Nurse Gillespie, it is much to befeared, was only the type of a class, and that class a numerous one. She was an ignorant woman, naturally deficient in the higher attributes of humanity, a sense of justice, patience, and kindness; and she was placed in a position requiring great patience, the exercise of a continual sense of justice, and infinite kindness of heart. Under the demands of her position the little humanity she had gave way, and she set about ruling her small kingdom by the ordinary methods of the vulgar despot; kicks, cuffs, foul words terrorisation of every conceivable kind were the means she used for producing ready submission to her will and for securing as easy a life for herself as she could compel her circumstances to yield her. Ignorant selfishness was probably the main factor in her character But there are, undoubtedly, scores and hundreds of Nurse Gillespies in similar situations. That is the terrible significance of her exposure. Hundreds perhaps thousands, of miserable little victims are now enduring daily agonies and terrors at the hands of "nurses"—save the mark—as selfish and as entirely ignorant as Nurse Gillespie. It is the system which is at fault. This is the point we desire to insist upon. Ignorant people cannot rule; least of all can they rule children. For the management of little ones education and high natural character and capacity are indispensable. This is what the average Guardian can never understand. It is what our civilisation must compel him to understand. We need an apostle for workhouse children; a female apostle if possible. The system of management must be altered. It must be altered in this way, that no nurses or other persons must be put into positions of authority who have not education and the capacity for control. That is the secret of the deliverance of the children of the workhouse. Whilst things continue as they are our Guardians are responsible for a system which lends itself to such daily torture, terror, and suffering among helpless children as constitute a mockery of the Christian religion and a scandal to civilisation.

### A "Bitter Cry" from Cornwall.

It is often taken for granted that modern England is the product of the combination of Norman with Saxon after the Roman occupation; and that if Cæsar had never landed on these shores the ancient Britons would still have been in the ascendant, and their skin costumes, with their Druidic rites, would have constituted the most picturesque, if the least civilised, features of our sea-girt isle. It might have been so, and it might not. Cornish people tell us that it would not. That they are ancient Britons they proudly admit; but that they prefer untanned skins to good leather, and venerable smells to the sweeter odours of cleanliness and civilisation they deny, and that with an energy which for the timid may even have its alarming side. On the contrary they claim to be in the very van and forefront of all that constitutes true civilisation. There are not wanting justifications for this high claim. Newquay is a Cornish town and seaport, which until quite recently the average Londoner had never heard of. But Newquay insists that she is highly civilised, and in proof thereof she sends up a petition to her County Council for one of the most characteristic outward and visible signs of advanced civilisation. In short, Newquay insists that Cornwall cannot and ought not to be "happy until she gets" a "medical officer of health for the county." In this we, at any rate, shall entirely agree with Newquay; for we hold, as we suppose every person who has even an elementary knowledge of sanitary science must hold, that no county can be considered to be anything but laggard and unscientific which has not "a county medical officer of health." Cornwall has a bishop all to herself, and she is justly proud of him. A county medical officer of health is the bishop of the bodies of the population of the county, and he is as entirely indispensable to all-round well-being as the bishop of souls. We add our prayer to that of the Newquay Local Board, and urge upon the Cornwall County Council the appointment of a medical officer of health for the county without delay. We go further and affirm that not only Cornwall but every other county in Great Britain must be considered dangerously backward in its civilisation until it appoints for itself, and itself alone, a county medical officer of health.

### Dry Sanitation.

It is delightful in medicine to get hold of a genuine practical enthusiast. So many members of our profession are mere "bookish prigs"—we write the words with fear and trembling—that a little bit of plain, honest, useful, and available practice is most stimulating. Our practical man for the moment is Dr. G. V. Poore, and his subject is "privies." Dr. Poore believes in privies; and so does the present writer, who was brought up in a lonely house in the country, where nothing but privies were available; and where a whitewashed privy in the garden, which was as clean within and without as newly-ironed bed-sheets, and which was cleared at very short intervals for manurial purposes, left absolutely nothing to be desired in the way of wholesome and pleasant country sanitation. Dry sanitation is Dr. Poore's gospel. The first thing to be done, in the way of privies, Dr. Poore tells us, is to cut them off from the house—put them outside its four walls. Approach them by a covered way, with cross ventilation, if you please; but outside of the house they must be. Make ample provision for the draining away of all fluids from the soil catch; expose the soil to air, and, if possible, to sunlight. Air and sunlight are nature's deodorisers. For æsthetic purposes a little earth may be used, but Dr. Poore assures us it is not necessary. Remove the soil frequently for the enrichment of the garden. This, in brief, is Dr. Poore's theory, and, we are glad to learn, his entirely successful practice. For country houses and villages it is admirable. We are sure it would be equally admirable for large towns, if we could but get engineers with as clear heads and as honest purposes as Dr. Poore's. Mr. Edward Stanford, of Cockspur Street, W., publishes Dr. Poore's expositions in detail in a shilling pamphlet.

